# BCI2000Web and WebFM: Browser-Based Tools for Brain Computer Interfaces and Functional Brain Mapping

**DOI:** 10.3389/fnins.2018.01030

**Published:** 2019-02-13

**Authors:** Griffin Milsap, Max Collard, Christopher Coogan, Nathan E. Crone

**Affiliations:** ^1^Department of Biomedical Engineering, Johns Hopkins University, Baltimore, MD, United States; ^2^Department of Neurology, Johns Hopkins Universit, Baltimore, MD, United States

**Keywords:** electrocorticogram (ECoG), functional brain mapping, visualization, web browser, brain computer interface (BCI)

## Abstract

BCI2000 has been a popular platform for development of real-time brain computer interfaces (BCIs). Since BCI2000's initial release, web browsers have evolved considerably, enabling rapid development of internet-enabled applications and interactive visualizations. Linking the amplifier abstraction and signal processing native to BCI2000 with the host of technologies and ease of development afforded by modern web browsers could enable a new generation of browser-based BCIs and visualizations. We developed a server and filter module called BCI2000Web providing an HTTP connection capable of escalation into an RFC6455 WebSocket, which enables direct communication between a browser and a BCI2000 distribution in real-time, facilitating a number of novel applications. We also present a JavaScript module, bci2k.js, that allows web developers to create paradigms and visualizations using this interface in an easy-to-use and intuitive manner. To illustrate the utility of BCI2000Web, we demonstrate a browser-based implementation of a real-time electrocorticographic (ECoG) functional mapping suite called WebFM. We also explore how the unique characteristics of our browser-based framework make BCI2000Web an attractive tool for future BCI applications. BCI2000Web leverages the advances of BCI2000 to provide real-time browser-based interactions with human neurophysiological recordings, allowing for web-based BCIs and other applications, including real-time functional brain mapping. Both BCI2000 and WebFM are provided under open source licenses. Enabling a powerful BCI suite to communicate with today's most technologically progressive software empowers a new cohort of developers to engage with BCI technology, and could serve as a platform for internet-enabled BCIs.

## 1. Introduction

A brain-computer interface (BCI) is a system that translates brain activity into control signals for a computer. Modern incarnations of BCIs rely on rapid and low-latency brain signal acquisition, preprocessing, feature extraction, classification and/or regression, and frequently, postprocessing of the resultant control signal (Wolpaw et al., 2002). In the case of closed-loop BCI, some form of visual or auditory feedback is given to the user to inform them of their control performance, typically requiring a low round-trip latency from signal acquisition to output. BCI development typically requires performant implementations of data acquisition and signal processing algorithms, high precision synchronization of external device telemetry, and typically, control of external software, requiring inter-process control or device input emulation (Wolpaw et al., 2000; Vaughan et al., 2003).

These technical requirements make the development of software for this purpose extremely challenging; however, there are a number of existing software platforms that bootstrap this development endeavor. BCI2000 has been a standardized research platform for BCI development for the last 15 years; it has been used by over 400 labs, and has been cited in numerous publications (Schalk et al., 2004). OpenViBE is another platform that has been developed to support real-time BCI research, offering a graphical programming language for signal processing and visualization (Renard et al., 2010). Additionally, a low-level communication protocol supporting signal acquisition and synchronization, called LabStreamingLayer, allows for TCP network streaming and synchronization of multi-modal data streams (Kothe, 2016) and could form the foundation of a BCI platform.

Widespread adoption and advancement of web browser technology makes it an attractive target for a BCI platform. Recent advancements in browser technology and standards have enabled direct access to low-level system resources such as graphics hardware and accelerometry/system sensors with application programming interfaces (APIs) that have exposed this hardware and software functionality via easy-to-use yet powerful and performant JavaScript packages. Network-enabled services also implement publicly available APIs that allow developers to call upon remote computational resources, such as Amazon web services (AWS), or to query information from vast databases of indexed knowledge, such as Wikipedia and Google Image Search. Moreover, many libraries supporting visual presentation of user interfaces and data visualizations have been developed. For example, d3.js (Bostock, 2011) has been used to power interactive data visualizations with impressive performance and an expressive yet functional API.

Many of the technologies readily available in the modern web browser would be useful to have available for the development of a contemporary BCI—for example, the ability to tag data in real-time with a speech transcription, via the WebSpeech API (Shires and Wennborg, 2012), or the ability to present stimuli in 3D using a virtual reality headset, via WebVR (Vukicevic et al., 2016) and three.js (Cabello et al., 2010). Visualization of the resulting data using d3.js (Bostock, 2011) or even sonification using the WebAudio API (Adenot et al., 2018) are fruitful endeavors for understanding realtime BCI output. Existing BCI software suites generally provide some amount of interprocess communication, typically exposed via user datagram protocol (UDP) or shared memory. However, browsers do not typically allow web apps to access UDP natively due to security concerns; further, existing communication schemes like BCI2000's AppConnector interface do not scale well to high data volumes, like those required to transmit human electrocorticography (ECoG) signals. BCI2000's existing interprocess communication tooling was designed with the transmission of control signals in mind, communicating signals using ASCII for simplicity instead of binary at the expense of inflating the data rate by a factor of ~8-fold—an approach that was successful until the need to transmit raw and processed ECoG data streams was desirable. Modern browsers implement a protocol built on top of TCP called WebSocket (Fette, 2011) that allows an HTTP client to escalate an existing connection to a general purpose real-time bidirectional binary/ASCII communication interface. WebSockets are perfectly situated to facilitate the transfer of raw brain signals, extracted neural features, and processed control signals from a BCI software suite to a web app on a browser-enabled device, as well as the transfer of auxiliary sensor information from the web app back to the native software suite, all in real time. In this article, we present an implementation of the aforementioned interface as a plugin to BCI2000, which we call BCI2000Web.

### 1.1. ECoG Functional Mapping: A Testbed for Web Technologies

In this report, we additionally demonstrate the utility of this new BCI2000Web interface with an example application that shares many technical requirements with a BCI: a functional mapping tool capable of visualizing cortical activation derived from ECoG recordings in real-time using local processing at the bedside or in the operating room, and of synchronizing the final results to a centrally hosted repository.

Functional mapping of eloquent cortex is a target application of great scientific and clinical impact. About a third of patients with epilepsy have seizures that are resistant to medication therapy. In many of these patients, seizures arise from a focal brain area, and if this area can be safely removed, seizure control can be achieved. When non-invasive testing cannot reliably identify the seizure onset zone as distinct from brain regions needed for normal neurological function, clinicians may choose to surgically implant electrodes in the depths of the brain (stereo-EEG) or on its surface (electrocorticography, or ECoG). These intracranial electrodes may be implanted for a week or more in order to reliably localize the onset of seizures. These electrodes also facilitate the identification of eloquent cortex—i.e., regions that are implicated in speech and language, as well as perception, movement, and other important brain functions. A technique called electrocortical stimulation mapping (ESM) is typically used to map these regions. During ESM, pulse-trains of electrical current are passed between pairs of the implanted electrodes to temporarily disable a small patch of cortex while the patient performs a simple language or motor task. A behavioral change elicited by this temporary lesion indicates that the stimulated area of the brain is necessary for task completion (Ojemann et al., 1989). This testing procedure is time-consuming and uncomfortable for the patient, sometimes eliciting after-discharges (Lesser et al., 1984; Blume et al., 2004); these after-discharges can also evolve into seizures, which can be of questionable utility for diagnosing ictal cortex (Hamberger, 2007).

The limitations of ESM have motivated a complementary mapping technique based upon estimates of task-related changes in the power spectra, especially in high frequencies, of passive recordings of ECoG or stereo-EEG during behavioral tasks. This mapping technique, hereafter referred to as ECoG functional mapping, produces maps of task-related cortical activation, which may include cortex that is recruited by a task but not critical to task performance. In contrast, ESM uses a temporary electrophysiological disruption of cortical function to simulate the acute behavioral effects of tissue resection, and is presumed to be specific to areas critical to task performance. Nevertheless, a number of clinical studies have demonstrated good correspondence between ECoG functional mapping and ESM (Brunner et al., 2009; Wang et al., 2016). Moreover, several studies have shown that ECoG functional mapping can be used to predict post-resection neurological impairments, and in some cases it has predicted impairments that were not predicted by ESM (Wang et al., 2016). For these reasons, some epilepsy surgery centers have begun to use ECoG functional mapping as a complement to ESM, sometimes providing a preliminary map of cortical function that guides the use of ESM. However, most epilepsy centers have not yet adopted ECoG functional mapping because of the lack of technical resources, especially software that can be used with their clinical EEG monitoring systems.

Several ECoG functional mapping packages have been developed in recent years. For example, SIGFRIED acquires a large baseline distribution of neural activity in a calibration block, then rapidly accumulates estimates of cortical activation by averaging neural activity evoked by behavior in blocks of time (Brunner et al., 2009). A commercial product called cortiQ (Prueckl et al., 2013) is capable of performing this block-based mapping paradigm, which makes it possible for minimally trained clinical professionals to perform passive ECoG mapping. Both SIGFRIED and cortiQ are built using the BCI2000 framework and take advantage of the extensive optimizations and development legacy of the platform. A more nuanced mapping technique, termed spatial-temporal functional mapping (STFM), provides time-resolved, trial-locked results during a specific task by collecting a pooled baseline activity from a pre-defined 1~ s period before the onset of a trial, then performing a statistical test on each time/channel bin in a window of interest relative to trial onset (Wang et al., 2016). Though the results of STFM are more complicated and require more expertise to interpret than the block-based mapping used by SIGFRIED or CortIQ, they provide a more detailed map of the spatial-temporal evolution of task-related activation, which can help clarify the role of different areas activated by a given task, of clear utility in cognitive neuroscience research and of potential clinical utility in planning surgical resections.

ECoG functional mapping relies on high performance signal processing and sophisticated real-time visualization, making it a suitable application example for BCI2000 and BCI2000Web. We saw an opportunity to build an easy-to-deploy-and-use tool for both researchers and clinicians that delivers the time-resolved, trial-locked results of STFM at the bedside in a web application, using BCI2000Web as the underlying communication technology to drive a browser-based interactive visualization. As a demonstration of the potential of the BCI2000Web plugin, in this report we also present WebFM, a software suite built on top of Node.js and BCI2000Web for performing real-time functional mapping in a web browser.

## 2. Design and Implementation

We chose to build our BCI WebSocket interface on top of BCI2000 as opposed to the other aforementioned technologies for many reasons, including support for acquisition devices in common use within epilepsy monitoring units and EEG research lab settings, high performance spectral extraction implementations, pedigree within the research community, highly accurate stimulus presentation capabilities, comprehensive documentation, and its ability to replay experimental sessions *post hoc* easily and accurately.

**Figure 1:**
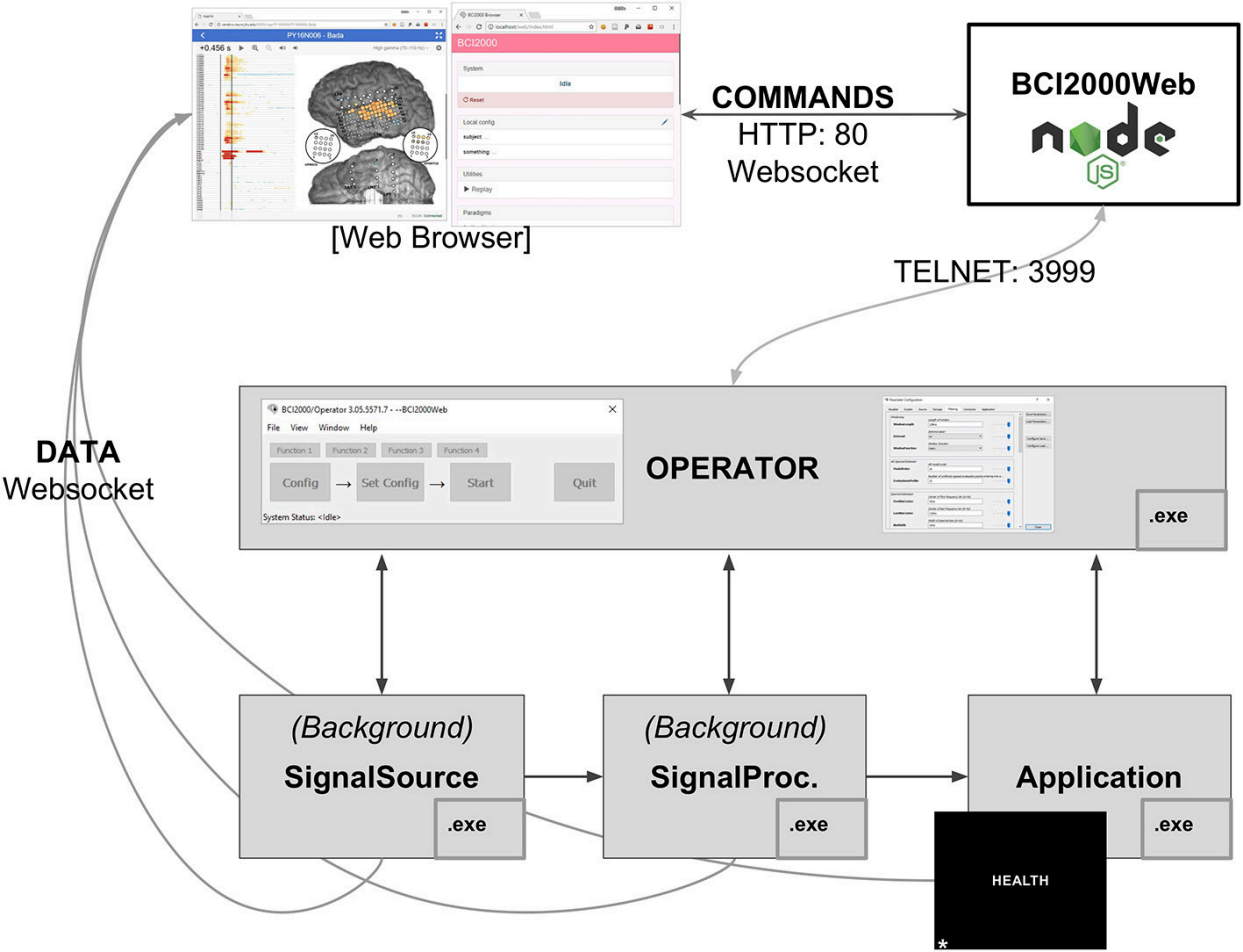
A full BCI2000 stack including a Signal Source, Signal Processing, Application, and Operator module communicates with BCI2000Web, implemented as a Node.js module, via Telnet. Browser-based remote control software and visualization tools interact with BCI2000Web, and receive raw and processed neural signals directly from the BCI2000 system modules, via WebSockets, while the application module presents stimuli to the patient, in this case, the word stimulus “HEALTH” for a word reading paradigm.

The BCI2000 environment is a general-purpose computational framework, typically used to construct BCIs, built upon four binary executables: the signal source module, which acquires physiological data from a supported amplifier; the signal processing module, which extracts neural features and transforms those features into control signals; an application module, which reacts to those control signals and provides feedback to the subject; and an Operator module, which orchestrates the behavior of all three functional submodules of the system (see Figure 1). Signals propagate from the source module to the processing module to the application module, with interconnections facilitated by a network-based protocol (in older versions of BCI2000) or a shared memory interface (in more recent iterations). Each of the modules consists of a series of signal “filters,” which accept an incoming signal (as a channels-by-elements array) and output a derived signal, potentially of different dimensionality. A built-in Operator scripting language allows for setup and configuration of filters within an experimental session to occur automatically, and a Telnet interface exists in the Operator module, capable of accepting textual commands in the Operator scripting language from outside BCI2000.

### 2.1. BCI2000Web

To address remote control of BCI2000 and data transmission between BCI2000 and browsers, we developed a Node.js module called BCI2000Web that accepts Operator scripting language commands via WebSocket and transmits them to the Operator executable via Telnet, returning system output back to the client. It is primarily used to control data acquisition and signal processing parameters remotely via a connected WebSocket-enabled client, typically a browser. BCI2000Web has been developed as a service that runs within the Node.js runtime. Upon starting, it opens a Telnet connection to the Operator module and also functions as a basic HTTP server. While BCI2000's Telnet implementation only supports one client sending one set of instructions that are executed serially, BCI2000Web provides an interface that allows multiple clients to make requests to send commands to the Operator module; these commands are queued and executed sequentially, with responses sent back to the appropriate client asynchronously. BCI2000Web is capable of interfacing with an unmodified BCI2000 distribution and automating system configuration without any further software or modifications to BCI2000 modules.

In order to transmit the raw and processed signal from the BCI2000 filter pipeline to the browser, however, source modifications within the system modules are required. The raw and processed signal is never sent directly to the Operator module, so the signal can only be transmitted to a browser by compiling secondary WebSocket servers into the existing modules at specific locations within the filter chain. This modification has been realized in our implementation as a generic “WSIOFilter” (**W**eb**S**ocket **I**nput/**O**utput Generic**Filter**) that can be instantiated multiple times into the BCI2000 filter chain. Each WSIOFilter defines a parameter specifying the address and port its WebSocket server is hosted on. Once an incoming connection is escalated to a WebSocket, this filter sends packets to the client in the BCI2000 binary format, first describing the dimensionality of the signal and the system state vector via a “SignalProperties” and “StateList” packet, then a “GenericSignal” and “StateVector” packet for the current system signal and state vector once per sample block. These filters can be instantiated several times in the signal processing chain for any particular signal processing module. This filter has also been included as a source module extension that enables transmission of the raw signal in all signal source modules, and an application module extension that enables transmission of the application module input—identical to the signal processing output—in all application modules. In practice, the amount of data being sent/received by instantiations of the WSIOfilter is directly related to CPU usage on the sending and receiving machines, while the latency of system throughput from recording to browser is more a function of the network setup and the number of network interface hops the data has to traverse.

A WebSocket-enabled client is unlikely to natively understand the format of the incoming/outgoing messages on any of the aforementioned connections: our implementation of BCI2000Web adds some decorators to Operator scripting commands and Operator outputs to handle multiple clients, and the WSIOFilter output is implemented in the BCI2000 binary protocol. A JavaScript library, bci2k.js—available as a package on the Node package manager (NPM) registry—contains functions that manage the BCI2000 WebSocket connections and translate the binary BCI2000 format into readily usable data structures within a JavaScript context. Non-browser WebSocket-enabled clients will need to implement this functionality in order to communicate using these interfaces.

### 2.2. WebFM: Browser-Based ECoG Functional Mapping

Subdural ECoG recordings are the target modality for WebFM, the aforementioned functional mapping application; this modality has different signal processing requirements than scalp EEG. The signal processing module used in the system in the Johns Hopkins Epilepsy Monitoring Unit is a modification of the default SpectralSignalProcessing.exe module. This signal processing module consists of a chain of filters, the first of which is a spatial filter capable of applying a common average reference, a frequently used spatial filter for ECoG recordings (Liu et al., 2015). This is followed by a series of IIR Butterworth filters, including a fourth order low pass at 110 Hz, followed by a second order high pass at 70 Hz and a 4th order notch filter at 60 Hz. After the signal is downsampled to 500 Hz from the native sampling rate, it is passed through a spectral estimator filter, which generates an autoregressive model on a window of filtered data and uses the model coefficients to form an estimate of the signal's power spectrum, using the Burg method (Burg, 1968). A WSIOFilter is instantiated at this point in the filter chain, capable of streaming this estimated spectral content of the neural signals in real-time. A system diagram and description of the system topology is detailed in Figure 1.

**Figure 2:**
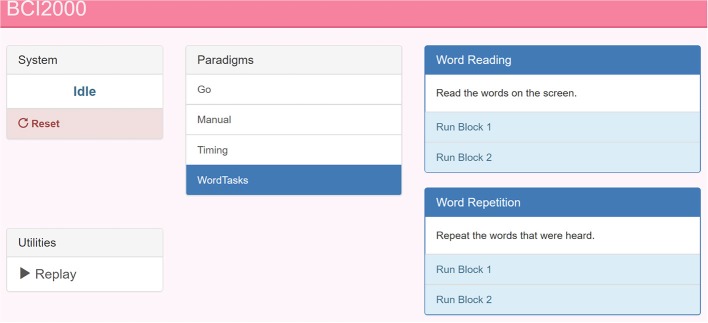
A screenshot of the BCI2000 remote control interface. The paradigm index is hosted by BCI2000Web over HTTP. This page is populated by the experimental paradigms present on the host machine (center) with buttons to start sub-tasks and specific blocks (right). A pane in the top left reads out the current BCI2000 system state, in addition to a system reset button. In the bottom left, a link to the system replay menu allows for recorded BCI2000 .dat file playback for system testing and offline mapping.

A language or motor task is parameterized as a BCI2000 .prm file and a collection of audio-visual stimuli in a git repository hosted on GitHub, available as packages that remote-control BCI2000 using the BCI2000Web server. Any number of these tasks can be checked out into the BCI2000Web distribution, and the server will automatically present them as startup options within the built-in BCI2000Web browser interface, shown and described in Figure 2. These paradigms typically specify a parameterization for StimulusPresentation.exe, a BCI2000 application module capable of presenting audio-visual stimuli to the patient with high-precision timing and sequence control. A browser is used to communicate to the bedside data-collection and stimulus-presentation machine, and to set up this system parameterization. (Because of this setup, it is notable that, when high-precision control isn't needed for stimulus presentation, the tasks presented to patients may themselves be interactive web applications, utilizing bci2k.js and BCI2000Web to inject behavioral markers into the data recorded by BCI2000.) A monitor and speaker connected to the bedside computer is set up in front of the patient, and a microphone is connected to the auxiliary analog inputs provided by the acquisition system, to be digitized synchronously with the electrophysiology.

The WebFM/BCI2000Web system currently supports more than 20 possible experimental paradigms, including a task battery used for clinical assessment for functional localization. These paradigms are currently versioned in GitHub repositories with group permissions and access control managed by the authors. A setup script is provided with BCI2000Web that accepts a GitHub login and clones/updates all available task repositories into the proper location.

#### 2.2.1. Patients and Electrode Localization

All aspects of this study were carried out in accordance with the recommendations of the Johns Hopkins Institutional Review Board with written informed consent from all subjects. All subjects gave written informed consent in accordance with the Declaration of Helsinki. The protocol was approved by the Johns Hopkins Institutional Review Board.

Before any functional mapping sessions occur with a patient, a post-operative computed tomography scan containing electrode locations is co-registered to a pre-operative magnetic resonance imaging scan of sufficient resolution (typically with voxel dimensions of 1 mm or less) to render the patient's cortical surface anatomy in high detail, using Freesurfer (Fischl, 2012) or Bioimage Suite (Papademetris et al., 2006). These electrode locations are overlaid on a 2D rendering of the cortical surface. An image file depicting this cortical anatomy and electrode layout, as well as a comma-separated value (.csv) file containing the normalized image coordinates of each electrode, is uploaded to the WebFM server via controls within the WebFM browser interface. This layout doesn't typically change during a patient's EMU stay, and it is referenced and retrieved by using a subject identification code, effectively de-identifying the reconstruction for research purposes.

#### 2.2.2. Software

**Figure 3:**
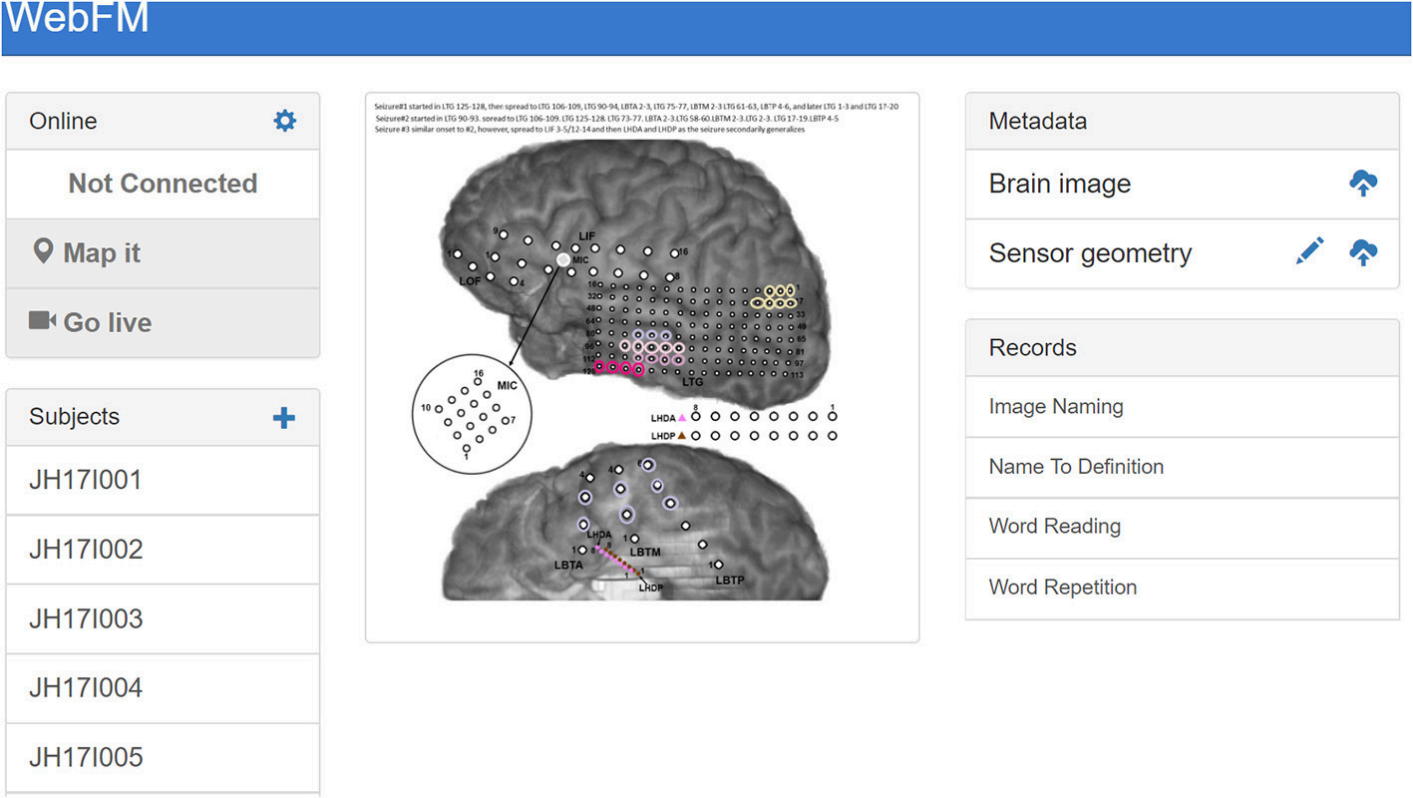
The landing page for WebFM. A pane in the top left shows system state and houses buttons that start trial-based functional mapping paradigms and a “live” mode that visualizes neural activity on the brain in real-time, as visualized in prior studies (Lachaux et al., 2007). A list of subject identifiers on the bottom left pane enables users to pull up previous/current subjects; a list of saved maps for the selected subject appears in the “Records” pane on the bottom right. The “+” in the top left of the “Subjects” pane allows operators to add new subjects to the database, and the “Metadata” pane at the top right allows operators to upload brain reconstruction images and normalized electrode locations for displaying functional mapping results. The brain images used for mapping are often overlaid with information about seizures and/or ESM results, so that functional activation can be easily visually compared with these data; the image shown in the center includes colored circles depicting the hypothesized spread of ictal activity during the subject's seizures.

During an ECoG functional mapping session, a browser running on the visualization device contacts the WebFM server and queries the bedside machine for the subject's identification code and what task is currently running. The WebFM server then serves the corresponding cortical reconstruction image and sensor location file in addition to a bolus of javascript code that is capable of opening WebSockets to the BCI2000Web server and WSIOFilters running on the bedside machine. The code also contains statistics packages and graphical libraries necessary for acquiring, analyzing, and visualizing the data. The browser then opens these data streaming WebSockets and performs the mapping without further contacting the WebFM server. After each trial of the task, the visualization is updated and once a full task run has been collected, the resulting map can be saved back to the WebFM server for indexing and *post-hoc* inspection, available on the WebFM Landing page, detailed in Figure 3.

**Figure 4:**
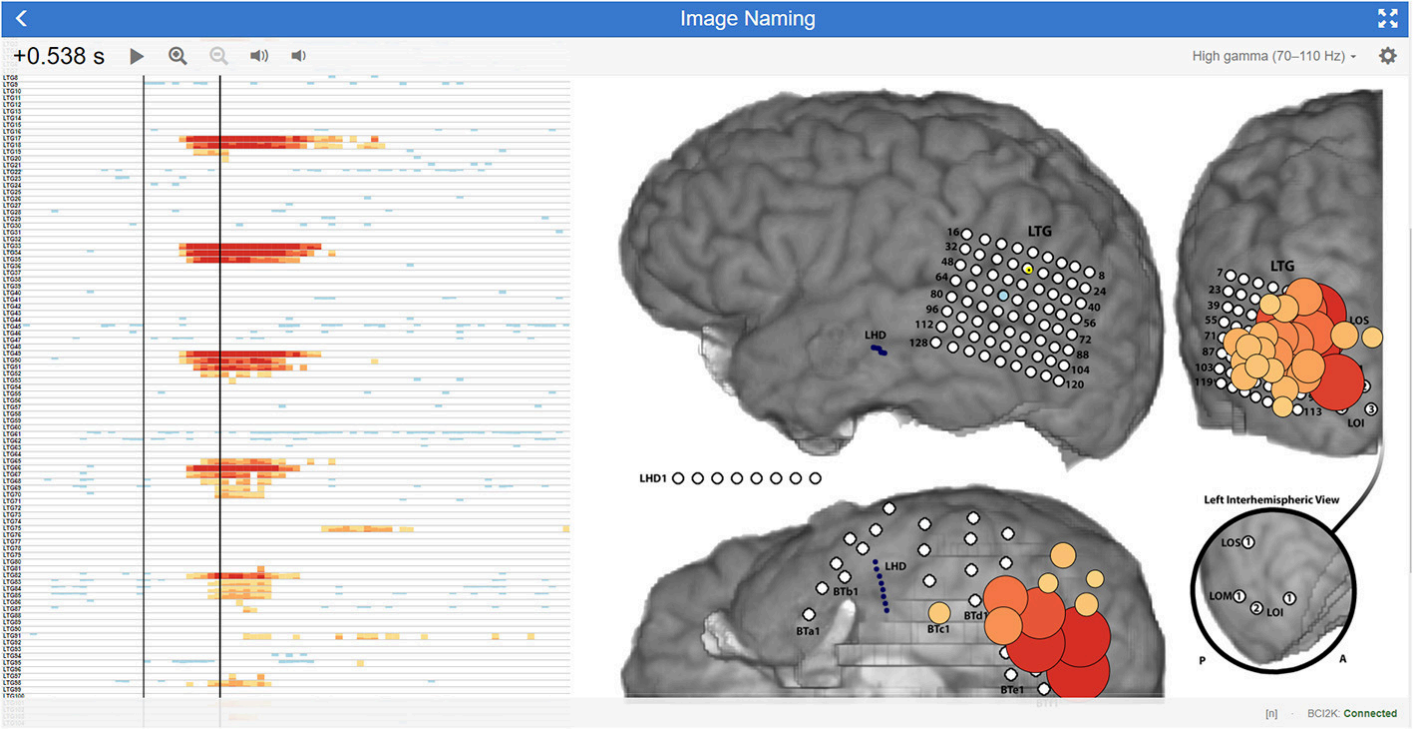
WebFM visualization description An example of WebFM results for an image naming task in a subject with high density (5-mm spacing) temporal-parietal-occipital electrode coverage. A horizon raster Heer et al. (2009) to the left shows a time (*x*-axis) by channels (*y*-axis) plot of trial-averaged task-modulated high gamma power, thresholded for statistical significance with BH correction for a FDR of < 0.05. Warm colors represent a statistically significant increase in task-modulated high gamma power, while cool colors indicate a statistically significant decrease in task-modulated high gamma power. The left black vertical bar within the raster indicates the trial-start (*t* = 0 s) where StimulusCode transitioned from zero to a non-zero value, indicating that a stimulus was being displayed. The right black vertical bar is a temporal cursor that interactively tracks the user's mouse gestures; the current time it indexes is shown in the top left corner, 0.538 s after stimulus onset. Buttons next to the selected time manipulate visualization properties. The current temporal slice is visualized on the brain image (right) as circles with size and color indicating the magnitude of the *z*-score, with the same coloration as in the horizon chart. A button in the top right maximizes the display to occupy the full screen-space of the device; a gear icon next to the fullscreen icon presents a configuration dialog box containing options for saving results, changing visualization parameters, configuring realtime signal or BCI2000 state trial-triggering, and visualizing the raw signal, amongst much more functionality. A drop down menu next to the gear icon turns on/off multiple visualization layers, enabling/disabling display of ESM, functional mapping, connectivity metrics, evoked responses, etc. A status message at the bottom right indicates WebFM has connected to BCI2000Web via bci2k.js and a trial counter, in this image represented with an “[n]”, increments as trials are delivered to and visualized by WebFM.

The statistics and visualization for WebFM are based on the techniques and methods described in Wang et al. (2016). The baseline window for the tasks is defined as a configurable period from 1,000 to 200 ms before the trial onset and a baseline distribution is formed per channel from the pooled high gamma power values during this period. A two-way *t*-test is performed between the distribution for each time-channel bin and that channel's baseline distribution. The resulting *p*-values are corrected for multiple comparisons using the Benjamini-Hochberg (BH) procedure, controlling the false discovery rate at 0.05 (Benjamini and Hochberg, 1995). This correction is used to threshold the results displayed in the WebFM raster and spatial plots: time-channel bins that did not survive the BH correction are hidden from view. Any individual time point in this raster can be dynamically selected and visualized by “scrubbing” the mouse cursor over the raster display; this yields circles drawn on a two dimensional representation of the electrode montage, highlighting which cortical locations were active during that particular time-point across trials. An options dialog allows users to change baseline periods, modify visualization timing parameters and amplitudes, as well as make comparisons across task conditions and contrasts. The visualization is shown and further described in Figure 4.

The visualization APIs exposed by WebFM can be used to implement a number of other visualizations as well. One mode of WebFM provides a visualization of raw high gamma activation in real time, as in (Lachaux et al., 2007); other modifications have also been used to visualize the propagation of interictal spiking and seizure propagation across cortex.

### 2.3. Deployment

As of the time of writing, the WebFM system has been deployed at two sites: the Johns Hopkins Hospital and the University of Pittsburgh Medical Center. Across these sites, WebFM has been used with three acquisition devices: the NeuroPort system (Blackrock Microsystems, Salt Lake City, UT), a Grapevine system (Ripple, Salt Lake City, UT), and the EEG1200 system (Nihon Kohden, Tomioka, Japan). Between these sites and amplifiers, WebFM has been used to create over 200 functional maps across 33 subjects. The majority of these subjects (19) were hospital inpatients undergoing epilepsy monitoring prior to resective surgery. Clinical staff in the Johns Hopkins Epilepsy Monitoring Unit have a link to the WebFM portal on their desktop machines and frequently use the passive ECoG mapping results when discussing surgical plans. The remaining 14 subjects were temporarily implanted with a 64-channel high density ECoG strip during lead implantation for deep brain stimulation; for these subjects, WebFM was used to map sensorimotor cortex in the operating room. WebFM has even been used to generate maps of activity recorded at one site by researchers at another site in real time, utilizing virtual private networks.

## 3. Discussion

BCI2000Web and WebFM take advantage of several recent technological developments. First and foremost, these packages capitalize on advancements in the modern web browser, which is quickly becoming a platform capable of general purpose computing. With a focus on frontend user interaction, many packages have been written in JavaScript that support the rapid implementation of interactive applications and visualizations. WebFM in particular makes use of d3.js (Bostock, 2011) to provide a high-quality interactive visualization of trial-averaged high gamma modulation directly on the brain.

**Figure 5:**
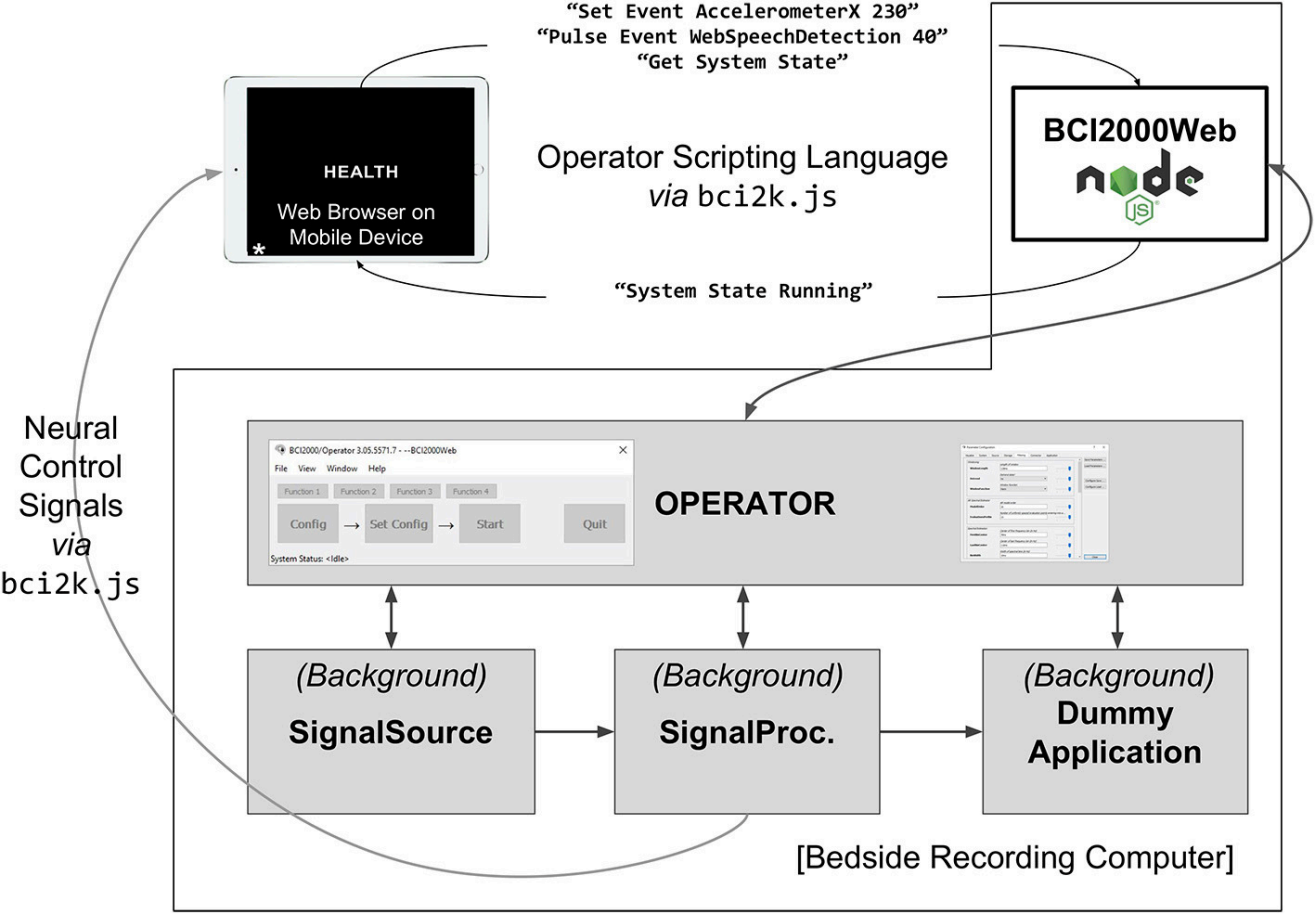
A system diagram depicting an experiment implemented in browser JavaScript running on an independent mobile device. The mobile device is running an experimenter-implemented web-page in fullscreen mode which communicates directly with BCI2000Web for event logging as well as the Signal Processing module for receiving extracted neural control signals. A JavaScript package, bci2k.js, manages WebSocket connections that handle transmission of operator scripting language commands and decodes neural control signals from a binary format. The mobile device is running a word reading paradigm (with the stimulus “HEALTH” currently presented) that has defined asynchronous experimental states including markers for automated vocal transcription onsets using the WebSpeech API. A query for system state is also relayed by the BCI2000Web server. The benefit of such an architecture is that the patient interface is separated from the bedside clinical acquisition machine and can be left with the patient without concern of the patient manipulating the clinical datastream.

The key to taking advantage of these web-based technologies is the implementation of BCI2000Web, which utilizes the WebSocket API to transmit binary-formatted brain data directly to the browser over TCP/HTTP, and which allows direct communication to and from BCI2000. While the experimental paradigms presented in conjunction with WebFM utilized the native BCI2000 stimulus presentation module to interact with the subject, the general-purpose access to Operator scripting over WebSockets provided by BCI2000Web easily lends itself to a different system architecture, in which a browser application itself is responsible for interacting with the subject and providing experimental markers sent via WebSocket; this topology is depicted in Figure 5. Several paradigm packages for BCI2000Web leveraging this architecture have been authored to date. Some make use of the WebSpeech API (Shires and Wennborg, 2012) to do real-time speech tagging and segmentation for tasks involving freely generated speech; another uses the WebMIDI and WebAudio APIs (Wyse and Subramanian, 2013) to register subject input on musical peripheral devices, and perform high-performance audio synthesis in response. Public JavaScript APIs allow for rich BCI interactions, and experimental paradigms can pull upon web resources such as Google Image search for providing varied and tailored stimuli at run-time. Extending this idea, it is easy to envision a system architecture in which users' neural data is sent to a browser application that communicates with a server backend in real time, allowing cloud-based services to apply sophisticated machine learning techniques that wouldn't be feasible otherwise on the client-side. Even further, one could develop a browser-based application that transmits multiple users' neural data to each other's clients, facilitating brain-based communication.

Cross-device compatibility is another advantage to using the browser as a visualization and stimulus presentation platform. Any browser-enabled device (smartphone, tablet, PC, or even game console) can be used to present stimuli or visualize output. Because of this “write-once, run-anywhere” development process, WebFM can be used by clinicians to view mapping results in real-time on their smartphones from outside the patient's room while ECoG functional mapping is being run by technicians.

### 3.1. Drawbacks and Caveats

The rationale behind the division of processing using native binaries and visualization using browser-interpreted javascript is due to current limitations inherent to browsers. Browser-hosted JavaScript is rapidly advancing as a next-generation efficient computational platform with the advent of WebAssembly and ASM.js (Herman et al., 2014), but at the time of writing it is still too computationally demanding to perform real-time feature extraction and signal processing in the browser. Furthermore, browser access to low level computer hardware and connected USB devices is only in the early development stages. Given these limitations, BCI2000Web was designed to take advantage of the device driver access and computational efficiency of the C++ code base that powers BCI2000 for acquisition device abstraction and signal processing/feature extraction. This architecture frees frontend developers from dealing with complicated signal processing code in JavaScript, and instead enables them to focus on user experience and design. In the future, a full-stack BCI2000 analog could be implemented directly within the browser, and BCI2000Web is a glimpse of what that software could empower for web developers with access to neural features.

A significant amount of the development effort for BCI2000 has been spent on implementing high-performance signal processing and stimulus presentation software. Delivering audio-visual stimuli to subjects with a consistent yet minimal latency is a non-trivial task that BCI2000 has accomplished by interfacing with low-level graphics drivers in a nuanced way. Operating system version, bit-width (32 vs. 64), driver versions, compiler optimizations, and varying hardware capabilities collude to make this stimulus presentation problem a fragmented and moving target—one which BCI2000 has historically hit with surprising accuracy, achieving visual presentation latency on the order of one to two frames at a 60 Hz monitor refresh rate and audio latencies on par with modern audio production software (Wilson et al., 2010). The BCI2000 core team encourage developers to implement custom signal processing and stimulus presentation paradigms within this BCI2000 environment using documented C++ code templates in order to benefit from these optimizations. That said, so long as tasks are designed properly and ground truth stimulus and response signals are collected (e.g., screen mounted photodiodes and patient facing microphones connected directly to auxiliary inputs on the amplifier), it is still possible to collect data of high scientific quality using the browser as the primary stimulus presentation software even if its stimulus display and communication latency are in question.

We benchmarked the visual timing performance of a system with and without BCI2000Web modifications using the procedure in Wilson et al. (2010) on a platform comprising Windows 7 64 bit with BCI2000 r5688, Google Chrome 67.0.3396.99, and a 256 channel 1,000 Hz recording from a Blackrock NeuroPort running with a 20 ms sample-block size; a standard configuration for a moderate-to-high channel-count ECoG recording running on an up-to-date clinical machine as of the time of writing. An unmodified BCI2000 distribution on this system exhibits a visual latency (*t*_3*v*_, as expressed in Wilson et al., 2010) of 52 ms with a standard deviation of 8.0 ms. With BCI2000Web sending neural signals to a browser via WebSocket on the same acquisition machine, a mean visual latency of 60 ms with a standard deviation 9.4 ms was observed. Using the hospital wireless network to send neural signals via WebSocket to a tablet PC running Windows 10 and the same version of Chrome results in a visual latency of 62 ms with a standard deviation of 13.4 ms. These latency metrics indicate a minimal impact to timing performance when using BCI2000Web. In many real-time BCI implementations, spectral feature extraction occurs in windows of 128–256 ms with a slide of 16–32 ms, and single-trial visual timing differences fall well within one windowing period. BCIs reliant upon time-domain features—in particular those that perform trial-averaging of evoked response potentials—will be more sensitive to these latency differences, and it is critically important to run timing benchmarks for specific hardware/software/network configurations in these circumstances. It should be noted that these performance metrics are configuration-specific and are likely to vary significantly across use cases; BCI2000Web comes bundled with an A/V timing paradigm that can be used to collect timing-test data, but analyses of these latencies and interpretation of what constitutes sufficient performance is application specific and is left to the end-user.

## 4. Conclusions

The development of a communication protocol that connects one of the most widely adopted BCI research and development suites with the power of modern browser technologies is expected to accelerate the pace of development for BCI technologies. Newer software developers, primarily taught using these modern software development paradigms, can now develop new BCI applications and neural signal visualizations while leveraging the legacy and performance of native BCI2000 modules. We have developed and presented a web-based ECoG functional brain mapping tool using this technology, and we have successfully deployed it at two sites with a cohort of 33 patients over two years. BCI2000Web and WebFM together utilize the relative strengths of a highly optimized C++ code base in BCI2000 and the high level visualization libraries within modern browsers to demonstrate a clinically useful and modern functional mapping tool. We have also used BCI2000Web for ongoing, albeit unpublished, BCI research projects, and we describe herein the advantages and potential uses of BCI2000Web in future BCI applications. This software is documented and released under permissive free and open source software licenses, and is put forward by the authors for use in the research and development of BCIs and multi-site studies on the clinical efficacy of ECoG functional mapping.

## Data Availability Statement

A standalone distribution of BCI2000Web is available on GitHub (github.com/cronelab/bci2000web). This distribution comes packaged with pre-compiled BCI2000 binaries that contain WSIOFilter taps for data access. The bci2k.js package—which translates BCI2000 binary packets from signal taps to usable data structures, and handles the Operator scripting language protocol—can be installed with NPM (node install bci2k); its codebase is available on GitHub (github.com/cronelab/bci2k.js). WebFM can also be found on GitHub (github.com/cronelab/webfm). All of this software is available under free and open source licenses.

The data used in the live demo at www.webfm.io is available via the WebFM API: the subject's brain image, base-64 encoded, is located at www.webfm.io/api/brain/PY17N009; the subject's sensor geometry is located, in JSON format, at www.webfm.io/api/geometry/PY17N009; the high gamma activation data for the presented task (syllable reading) is located at www.webfm.io/api/data/PY17N009/SyllableReading.”

## Author Contributions

BCI2000Web was developed by GM with assistance from MC. Testing and validation of BCI2000Web was performed by CC. WebFM was developed by MC with assistance and maintenance by CC and supervision by NC. Deployment and testing of WebFM in the Johns Hopkins Epilepsy Monitoring Unit was undertaken by GM, MC, and NC. This report was prepared by GM and MC, with input from NC.

### Conflict of Interest Statement

The authors declare that the research was conducted in the absence of any commercial or financial relationships that could be construed as a potential conflict of interest.

## References

[B1] Adenot P. Raymond T. Wilson C. Rogers C. (2018). Web Audio API Specification, W3C Candidate Recommendation. Available online at: https://www.w3.org/TR/webaudio/

[B2] Benjamini Y. Hochberg Y. (1995). Controlling the false discovery rate: a practical and powerful approach to multiple testing. J. R. Stat. Soc. B Methodol. 57, 289–300.

[B3] Blume W. T. Jones D. C. Pathak P. (2004). Properties of after-discharges from cortical electrical stimulation in focal epilepsies. Clin. Neurophysiol. 115, 982–989. 10.1016/j.clinph.2003.11.023 15003782

[B4] Bostock M. Ogievetsky V. Heer J. (2011). D^3^ data-driven documents. IEEE Trans. Visual. Comput. Graph. 12, 2301–2309. 10.1109/TVCG.2011.185 22034350

[B5] Brunner P. Ritaccio A. L. Lynch T. M. Emrich J. F. Wilson J. A. Williams J. C.. (2009). A practical procedure for real-time functional mapping of eloquent cortex using electrocorticographic signals in humans. Epilepsy Behav. 15, 278-86. 10.1016/j.yebeh.2009.04.001 19366638 PMC2754703

[B6] Burg J. P. (1968). A new analysis technique for time series data, in Paper Presented at NATO Advanced Study Institute on Signal Processing (Enschede).

[B7] Cabello R. (2010). Three.js. Available online at: https://github.com/mrdoob/three.js.

[B8] Fette I. Melnikov A. (2011). The Websocket Protocol. No. RFC 6455.

[B9] Fischl B. (2012). FreeSurfer. Neuroimage 62, 774–781. 10.1016/j.neuroimage.2012.01.021 22248573 PMC3685476

[B10] Hamberger M. J. (2007). Cortical language mapping in epilepsy: a critical review. Neuropsychol. Rev. 17, 477–489. 18004662 10.1007/s11065-007-9046-6

[B11] Heer J. Kong N. Agrawala M. (2009). Sizing the horizon: the effects of chart size and layering on the graphical perception of time series visualizations, in Proceedings of the SIGCHI Conference on Human Factors in Computing Systems, CHI '09, (New York, NY: ACM), 1303–1312.

[B12] Herman D. Wagner L. Zakai A. (2014). asm.js-Working Draft. Available online at: http://asmjs.org/spec/latest/

[B13] Kothe C. (2016). Lab Streaming Layer. Available online at: https://github.com/sccn/labstreaminglayer

[B14] Lachaux J. P. Jerbi K. Bertrand O. Minotti L. Hoffmann D. Schoendorff B.. (2007). BrainTV: a novel approach for online mapping of human brain functions. Biol. Res. 40, 401–413. 18575675

[B15] Lesser R. P. Lüders H. Klem G. Dinner D. S. Morris H. H. Hahn J. (1984). Cortical afterdischarge and functional response thresholds: results of extraoperative testing. Epilepsia 25, 615–621. 6479112 10.1111/j.1528-1157.1984.tb03471.x

[B16] Liu Y. Coon W. G. Pesters A. d. Brunner P. Schalk G. (2015). The effects of spatial filtering and artifacts on electrocorticographic signals. J. Neural Eng. 12:056008 10.1088/1741-2560/12/5/056008 26268446 PMC5485665

[B17] Ojemann G. Ojemann J. Lettich E. Berger M. (1989). Cortical language localization in left, dominant hemisphere. An electrical stimulation mapping investigation in 117 patients. J. Neurosurg. 71, 316–326. 2769383 10.3171/jns.1989.71.3.0316

[B18] Papademetris X. Jackowski M. P. Rajeevan N. DiStasio M. Okuda H. Constable R. T. (2006). BioImage suite: an integrated medical image analysis suite: an update. Insight J. 2006:209. 25364771 PMC4213804

[B19] Prueckl R. Kapeller C. Potes C. Korostenskaja M. Schalk G. Lee K. H.. (2013). cortiQ - Clinical software for electrocorticographic real-time functional mapping of the eloquent cortex, in 2013 35th Annual International Conference of the IEEE Engineering in Medicine and Biology Society (EMBC), 6365–6368. 10.1109/EMBC.2013.6611010 24111197

[B20] Renard Y. Lotte F. Gibert G. Congedo M. Maby E. Delannoy V. (2010). OpenViBE: an open-source software platform to design, test, and use brain–computer interfaces in real and virtual environments. Presence Teleoperat. Virt. Environ. 19, 35–53. 10.1162/pres.19.1.35

[B21] Schalk G. McFarland D. J. Hinterberger T. Birbaumer N. Wolpaw J. R. (2004). BCI2000: a general-purpose brain-computer interface (BCI) system. IEEE Trans. Biomed. Eng. 51, 1034–1043. 10.1109/TBME.2004.827072 15188875

[B22] Shires G. Wennborg H. (2012). Web Speech API Specification. W3C Community Group Final Report. Available online at: https://w3c.github.io/speech-api/

[B23] Vaughan T. M. Heetderks W. J. Trejo L. J. Rymer W. Z. Weinrich M. Moore M. M.. (2003). Brain-computer interface technology: a review of the second international meeting. IEEE Trans. Neural Syst. Rehabil. Eng. 11, 94–109. 12899247 10.1109/tnsre.2003.814799

[B24] Vukicevic V. Jones B. Gilbert K. Wiemeersch C. (2016). Webvr. World Wide Web Consortium.

[B25] Wang Y. Fifer M. S. Flinker A. Korzeniewska A. Cervenka M. C. Anderson W. S.. (2016). Spatial-temporal functional mapping of language at the bedside with electrocorticography. Neurology 86, 1181–1189. 10.1212/WNL.0000000000002525 26935890 PMC4818563

[B26] Wilson J. A. Mellinger J. Schalk G. Williams J. (2010). A procedure for measuring latencies in brain-computer interfaces. IEEE Trans. Biomed. Eng. 57, 1785–1797. 10.1109/TBME.2010.2047259 20403781 PMC3161621

[B27] Wolpaw J. R. Birbaumer N. Heetderks W. J. McFarland D. J. Peckham P. H. Schalk G.. (2000). Brain-computer interface technology: a review of the first international meeting. IEEE Trans. Rehabil. Eng. 8, 164–173. 10.1109/TRE.2000.847807 10896178

[B28] Wolpaw J. R. Birbaumer N. McFarland D. J. Pfurtscheller G. Vaughan T. M. (2002). Brain-computer interfaces for communication and control. Clin. Neurophysiol. 113, 767–791. 12048038 10.1016/s1388-2457(02)00057-3

[B29] Wyse L. Subramanian S. (2013). The viability of the web browser as a computer music platform. Computer Music J. 37, 10–23. 10.1162/COMJa00213

